# Fertility sparing surgery for ovarian sex cord stromal tumors: a nine case series

**DOI:** 10.11604/pamj.2018.31.221.15531

**Published:** 2018-12-05

**Authors:** Montassar Ghalleb, Hatem Bouzaiene, Sarah Sghaier, Hanen Bouaziz, Monia Hechiche, Jamel Ben Hassouna, Khaled Rahal

**Affiliations:** 1 Surgical Oncology Department, Institut Salah Azaiez de Cancérologie, Tunis, Tunisie; 2 Faculté de Médicine Tunis El Manar, Tunis, Tunisia

**Keywords:** Sex cord stromal tumors, ovarian cancer, fertility, surgery

## Abstract

Ovarian Sex Cord Stromal Tumors (SCST) are a rare disease carrying a good prognosis. They generally affect young women; therefore fertility preservation is a critical issue. Fertility Sparing Surgery (FSS) showed promising results in both oncologic safety and fertility preservation. A retrospective case series involving 9 patients diagnosed with SCST and treated with fertility sparing surgery at our institution was conducted between January 2000 and May 2015. The median age was 24 years old (10 to 39). The main clinical manifestation was pelvic pain seen in seven patients. Five patients complained about menstrual cycle disorders. The nine patients went through fertility sparing surgery; seven had conservative staging and the other two had a unilateral salpingo-oophorectomy. Three patients out of nine had a pelvic unilateral lymphadenectomy. Two patients received adjuvant chemotherapy. Only two patients presented locoregional recurrence that occurred respectively after 7 and 192 months. The treatment combined chemotherapy and surgery based on mass resection. One patient achieved a natural pregnancy after the treatment. FSS seems to be a suitable approach for SCST. However, more case series and meta-analysis should be conducted.

## Introduction

Sex cord stromal tumors (SCST) of the ovary are rare. They represent approximately 7% of all primary ovarian tumors [1]. SCST are considered as a low-grade disease [2] and their prognosis is associated with tumor grade and disease stage [3]. They generally occur in young women of reproductive age [4], raising the issue of fertility sparing surgery (FSS). Our aim is to report our experience of FSS in SCST of the ovary and discuss its feasibility.

## Methods

A retrospective case series involving 9 patients diagnosed with SCST and treated at our institution between January 2000 and May 2015. All the patients who had SCST with desire of pregnancy were included. The FSS consisted on the preservation of the uterus and the normal ovary with surgical staging; including: peritoneal cytology, omentectomy and multiple peritoneal biopsies. Lymphadenectomy was conducted only if there were suspicious lymph nodes. The FIGO 2014 ovarian cancer staging classification was used while writing this manuscript. The pathological diagnosis was made according to the international histologic classifications made by the World Health Organization (WHO). According to the decision of a multidisciplinary meeting, chemotherapy was indicated for all patients with IC tumors, tumor size >15 cm, poorly differentiated tumors and age inferior to 30 years.

## Results

The median age of our patients, was 23 years old (10 to 39). Four patients were married and two of them have already conceived. The median time for referral to the institute Salah Azaiez was 60 days (15 to 730). The main clinical manifestation was pelvic pain seen in seven patients (77%). Five patients (56%) complained about menstrual cycle disorders. Three (33%) had secondary amenorrhea and Two (22%) had menorrhagia. In the abdominal examination, three patients had palpable hypogastric mobile masses (33%). Five patients were virgin and refused vaginal touch. In the rectal touch a mobile painless mass in the Douglas pouch was identified in 2 patients. For the four other patients, the combined rectal and vaginal touch found a mobile left latero-uterin mass. All the tumors were viewed by ultrasound tests. Three of them (33%) were located in the right ovary, three (33%) in the left one and the last three (33%) were pelvic masses which could not be associated to the ovaries. The median size was 164 mm (56 to 400). Tumor markers such as; alpha-feto protein, beta-chorionic gonadotrophic hormone, CA125, CA19-9 and ACE; were measured in three patients (33%) and were normal as well as the hormones (estradiol, inhibin). Seven patients had conservative staging and the other two had a unilateral salpingo-oophorectomy. Three patients out of nine (33%) had a pelvic unilateral lymphadenectomy. All the lymph nodes were negative in the histologic examination. The median size in the histological examination was 150 mm (45 to 250) (Table 1). According to the decision of a multidisciplinary meeting, chemotherapy was indicated five patients. Three of them refused the systemic therapy. Two patients agreed and both had six courses of BEP. Chemotherapy was given every three weeks following surgery with no major side effects reported. One patient had a completion surgery three years after the initial surgery and one year after pregnancy. The median follow-up period was 45 months (13 to 195). Two patients (22%) presented loco regional recurrence that occurred respectively after 27 and 192 months. The first patient, with initially a poor differentiated tumor, relapsed with a 3cm mass in the pre-vesical peritoneum. She was treated with a complete mass excision followed by six courses of well tolerated BEP chemotherapy. The second patient relapsed in the retroperitoneum. The tumor was placed over aorta and intimately attached to the left renal vein, the tail of pancreas without invasion of these structures. The mass was dissected and fully removed. She had also six courses of BEP with no major side effects. One patient achieved a full term viable natural pregnancy two years after surgery (Table 2). Another patient had access to assisted reproductive technology, but the attempt failed and the pregnancy was not achieved.

**Table 1:** patient’s characteristics

Patients	Age	Suface	Tumor Size (mm)	Surgery	Controlateral ovary	Pelvic lymphadenectomy	Para-aortic lymphadenectomy	Definitive histologic examination	FIG
1	15	Normal	150	Conservative staging	No	No	No	Sertoli Leydig tumor	I A
2	33	Vegetation	100	Conservative staging	No	No	No	Sertoli Leydig tumor	I A
3	10	Normal	120	Conservative staging	No	Left	No	with annular tubules	I A
4	20	Normal	250	USO	Wedge biopsy	No	No	Granulosa tumor	I A
5	25	Normal	40	Conservative staging	No	No	No	with annular tubules	I A
6	20	Normal	170	Conservative staging	No	Right	No	Sertoli Leydig tumor	I C1
7	39	Normal	120	USO	No	No	No	Granulosa tumor	I A
8	20	Normal	150	Conservative staging	No	Right	No	Sertoli Leydig tumor	I A
9	34	Normal	400	Conservative staging	Wedge biopsy	No	No	Granulosa tumor	I A

*USO: Unilateral Salpingeco- Oophorectomy

* -: Not mentioned

*conservative staging: USO + Staging surgery (peritoneal cytology, omentectomy and multiple peritoneal biopsies)

**Table 2:** patient’s outcome

Patients	Chemo Therapy	Locoregional reccurence	Time to reccurence	Localisation	Treatment	Metastasis	Treatment	Pregnancy	Follow-up period	Death
1	No	No				No		No	25	No
2	No	No				No		Yes	25	No
3	No	Yes	192 months	Retroperitoneal	Surgery*+CT	No		No	192	No
4	Yes (BEP)	No				Yes	Flash irradiation	No	19	Yes
5	No	No				No		No	45	No
6	Yes (BEP)	No				No		No	55	No
7	No	No				No		No	53	No
8	No	Yes	27 months	Prevesical peritoneum	Surgery*+CT	No		No	50	No
9	No	No				No		No	13	No

*CT: Chemotherapy

*BEP: Bleomycin- etoposide- cysplatin

* surgery*: mass resection

## Discussion

The SCST of the ovary are rare tumors. They have better prognosis than the epithelial tumors [2]. Their prognosis is mainly associated with tumor differentiation and disease stage. They are most commonly diagnosed in women of reproductive age raising the issue of fertility preservation. The FSS is defined as the preservation of the uterus and an unilateral salpingo oophorectomy [2]. According to the American National Comprehensive Cancer Network (NCCN) 2017 guideline for ovarian cancer, the standard treatment for SCST stage IA/IC with fertility desiring is fertility-sparing surgery [2]. The necessity of a complete bilateral pelvic and para-aortic lymphadenectomy remains controversial [4]. Brown *et al.* (w) demonstrated that lymph node metastasis in ovarian SCST is rare. Therefore, there is no need for lymphadenectomy [4-6], unless clinically suspicious lymph nodes are present.

**Oncologic safety:** two out of nine patients recurred (22%) and were managed conservatively. To the best of our knowledge, no significant difference in disease-free survival or overall survival was found between the young patients with stage I who underwent a radical surgery and those who were subject to a less extensive surgery [2, 4, 7, 8]. In a recent retrospective, population-based cohort of 255 premenopausal women with SCSTs confined to the ovary, findings showed that patients who underwent FSS presented a cancer-specific survival that was inferior to those who conducted a definitive surgery (bilateral salpingo oophorectomy and hysterectomy [9]. However, this inferiority was shown after 20 years of follow-up. In the same study, there were no difference between the two groups of patients in overall survival [8]. Several recent studies, did not found a negative impact of FSS on recurrence or progression free survival [7, 9-14]. Although the role of adjuvant chemotherapy is not well established, NCCN recommends it in moderately or poorly differentiated SCSTs or SCST with heterologous elements [3]. It can also be given in SCST stage I with high-risk factors, such as tumor rupture, stage IC, tumor size >10-15 cm [2]. There is a lack of information about chemotherapy schemes, however BEP is frequently used [3]. In the series by Gui *et al*. DFS showed no significant difference between patients with moderately or poorly differentiated tumors whether chemotherapy was given or not. In the Mito-9 study [12], the adjuvant chemotherapy did not improve survival in stage Ic granulosa cell tumors. Besides, two patients with recurrence, were part of the group who had received chemotherapy. The use of completion surgery after pregnancy, or after the age of 40 years old is still debated, but can be considered in order to reduce the risk of recurrence on the spared ovary [14, 15].

Fertility outcome: SCST generally occur in women of childbearing age. Therefore, FSS is usually preferred to those of stage IA or IC wishing to retain fertility [2]. In some studies [2], biopsy of the normal ovary was not regularly conducted because of the risk of postoperative adhesions which can induce infertility or ovarian failure. Previous studies [2, 4], reported cases where pregnancy was achieved naturally or with assisted reproductive technology. Lee *et al.* [11], in their retrospective series of 36 patients 8 patients achieved 9 viable pregnancies. Ayhan *et al.* [14], in their study included 8 patient with SCST who underwent FSS found a fecundity rate of 40%. In the present study, one patient conceived naturally and another one was submitted to an assisted reproductive procedure which did not result in a viable pregnancy. Although information about the effect of chemotherapy on ovarian function is still subject to debate, the existing studies in the literature, did not succeed to determine the exact consequences of chemotherapy on the ovarian function [3]. It is known that, systemic treatment will reduce the ovarian reserve but it does not mean infertility [16]. Chemotherapy and fertility is a challenging topic for two reasons: first, is the scarcity of published cases in the literature and the second one it's the absence of a key determinant of fertility [17].

## Conclusion

SCST of the ovary are rare and have a wide range of symptoms. The treatment depends on the patient age, tumor differentiation and disease stage. As they generally occur in women of reproductively-active age, FSS for early stage (stage I) seems to be a safe approach provided that a close follow-up can be undertaken. However, due to the rarity of this disease, more case series and meta-analysis are required to back-up our findings and give a higher grade of recommendation of FSS in ovarian SCST.

### What is known about this topic

- Sex cord stromal tumors of the ovary generally affect young women; therefore fertility preservation is a critical issue;
- FSS is admitted for stage Ia and Ib in most histologic type, it is still controversial instage IC;
- More studies should be done for a higher grade of recommendation.

### What this study adds

- A retrospective case series involving 9 patients diagnosed with SCST and treated at our institution with FSS;
- Two patients presented a loco regional recurrence that occurred after 14 months. One patient out of eight (12.5%) achieved a natural pregnancy;
- FSS for early stage (stage I) seems to be a safe approach provided that a close follow-up can be undertaken.

## Competing interests

The authors declare no competing interests.
